# Research Note: Subzero saline chilling improved chilling efficiency and bacterial reduction of turkey carcasses

**DOI:** 10.1016/j.psj.2021.101458

**Published:** 2021-08-29

**Authors:** I. Kang, H.C. Lee, S.H. Park

**Affiliations:** ⁎ Departments of Animal Science, California Polytechnic State University, San Luis Obispo, CA 93407, USA; † Departments of Food Science & Technology, Oregon State University, Corvallis, OR 97331, USA

**Keywords:** subzero saline chilling, chilling efficiency, microbiological safety, turkey chilling

## Abstract

The poultry industry has attempted to improve carcass chilling efficiency, meat quality, and product safety. The purpose of this research was to investigate the effects of subzero saline chilling on carcass chilling time and microbial safety. Eviscerated tom turkeys were randomly picked from a local turkey processing plant and subjected to chilling in one of the 3 chilling solutions: 1) 0% NaCl/0.5°C (ice slurry control), 2) 4% NaCl/−2.41°C, and 3) 8% NaCl/−5.08°C. The turkey carcasses in subzero saline solutions were chilled more efficiently and reduced the chilling time over the carcasses in ice slurry solution. No significant difference was observed for carcass chilling yield and fillet cooking yield regardless of chilling method (*P* > 0.05). The number of mesophilic aerobic bacteria (**MAB**), *Escherichia coli* (***E. coli***), and total coliform cells were significantly reduced in the carcasses chilled in subzero saline solutions over the icy control, except MAB in 4% NaCl/−2.41°C (*P* < 0.05). Based on these results, the chilling of turkey carcass in subzero saline solution appears to improve carcass chilling efficiency and bacterial reduction, especially Gram-negative bacteria such as *E. coli* and total coliforms.

## INTRODUCTION

The United States is the world's largest turkey producer and exporter, and poultry consumers enjoy various turkey products for holiday dishes and day-to-day serving throughout the year. Possessing great nutritional values and superior health benefits, turkey products have been consumed for 7.3 kg (16.1 pounds) per person in 2019, which is doubled since 1970 (3.7 kg or 8.2 pounds) (National Turkey, 2021). In response to the increased demand, the average body weight of commercial meat-type turkeys increased from 5–7 kg in 1960s to ∼ 17 kg in 2000s in 16-wk age (Clark et al.., 2019). However, the faster growth and heavier muscles have shown lower product quality and more safety concerns especially when their carcasses are chilled slowly. It has been reported that PSE-like (pale, soft, and exudative) problems were observed in heavy turkey carcasses and/or muscles after slow chilling (McKee and Sams, 1998; Sosnicki and Wilson, 1991), whereas rapid chilling improved textural quality and bacterial reduction (Lee et al., 2016; Medellin-Lopez et al., 2014; Fernandez and Vierira, 2012; Savell et al., 2005). The incidence of human illness due to pathogenic bacteria in poultry products has not been reduced for the last 30 yr, and an innovative processing technology is highly required to improve food safety, product quality, and processing efficiency.

Recently, chilling of broiler carcasses in subzero saline solutions has been reported to improve food safety, product quality, and processing efficiency. After various tests using subzero saline solutions from 0% NaCl/0.5°C to 8% NaCl/−5.08°C, our laboratory observed that chilling of broiler carcasses in 4% NaCl/−2.41°C resulted in the best outcome for bacterial reduction, meat tenderness, and processing efficiency, with the potential savings of potable (or drinkable) water and wastewater (Kang, 2021; Kang, 2019; Metheny et al., 2019). However, no research has been conducted for turkey using the subzero saline technology. The purpose of this research was to evaluate the effects of subzero saline chilling (**SSC**) on turkey carcasses for chilling efficiency and bacterial populations.

## MATERIALS AND METHODS

All procedures were approved by the Institutional Animal Care and Use Committee of California Polytechnic State University (Protocol #2010).

### Brine Chilling Solution and Brine Ice Preparation

Three chilling solutions (0% NaCl/0.5°C, 4% NaCl/−2.41°C, 8% NaCl/−5.08°C) were prepared by adding ice in tap water for control and dissolving salt for saline solutions. Both saline solutions (4 and 8% NaCl, w/w) were then stored at −23°C to reach the target temperatures of −2.41 and −5.08°C, respectively, whereas the control solution was kept in a refrigerated room at 0.5°C. Extra solutions of the 3 treatments were frozen in Ziploc bags to maintain the target temperatures during carcass chilling.

### Turkey Carcass Processing and Chilling

A total of 12 eviscerated tom carcasses (2 carcasses/treatment for 2 replications; ∼ 30 kg/carcass) were randomly picked from the turkey processing line in a local turkey processing plant. They were immediately chilled using one of the 3 solutions: 1) 0% NaCl/0.5°C, 2) 4% NaCl/−2.41°C, and 3) 8% NaCl/−5.08°C. Before chilling, a thermometer was inserted to the center of breast fillet to monitor the internal temperature during chilling. During chilling, control ice and brine ice were added to maintain the target solution temperatures.

### Chilling Yield of Carcasses and Cooking Yield of Breast Fillets

After chilling, carcasses were hung on a shackle for 5 min, weighed, and evaluated chilling yield, using the formulation: (postchill carcass weight/prechill carcass weight) × 100%. For cooking yield, breast fillets were removed at 3-h postmortem and stored in a cooling room at 2.0°C for 24 h after inserting into individual Ziploc bags. In the following day, the fillets were weighed, placed on stainless steel racks in stainless trays, covered in foil, and cooked to an internal temperature of 75 to 78°C in a convection oven (36S-Y1A Wolf Challenger XL Range, ITW Food Equipment Group LLC, Gleenview, IL), according to USDA-Food Safety and Inspection Services guidelines (2001). The cooking yield was then calculated using the formulation: (post-cook weight)/(pre-cook weight) × 100.

### Microbiological Analysis

After chilling, 25 g of skin was aseptically taken from the breast area and placed in sterile WhirlPak bag. Each sample bag was stomached for 1 min after adding 225 mlLof sterile phosphate buffer saline (**PBS**).

• Mesophilic aerobic bacteria (**MAB**): Serial 10-fold dilutions of the stomached samples were surface-plated (0.1 mL) in duplicate on Petrifilm Aerobic Count Plates (3M Microbiology Products) to enumerate MAB after incubation at 37°C for 24 h.
• *Escherichia coli* (***E. coli***) and coliforms: Serial 10-fold dilutions of the stomached samples were similarly plated (0.1 mL) on Petrifilm *E. coli*/coliform count plates (3M Microbiology Products). All samples were incubated at 37°C for 24 h before enumeration.

### Statistical Analysis

Data in 4 replications were statistically analyzed by one-way ANOVA, using PASW 18 statistic program and a completely randomized design. A post-hoc analysis was performed using Duncan's multiple range test to evaluate differences among treatments (*P* < 0.05; SPSS, 2011).

## RESULTS AND DISCUSSION

During chilling, the internal temperatures of eviscerated carcasses continuously reduced from 42°C to 4.3–4.5°C in the most efficient way in 8% NaCl/−5.08°C, followed by 4% NaCl/−2.41°C and 0% NaCl/0.5°C solutions. Similarly, the chilling time of broiler carcasses was reported to reduce more effectively in 4% NaCl/−2.41°C than 3% NaCl/−1.8°C and 0% NaCl/0.5°C (water control) (Kang et al., 2021; Metheny et al., 2019). It has been known that rapid chilling of turkey improved protein quality and textural properties of their muscle, whereas delayed chilling could induce the development of PSE-like meat especially in heavy birds due to the protein denaturation by combining high temperature and low pH (Lee et al., 2019, 2014; Medellin-Lopez, et al., 2014; McKee and Sams, 1998). The economic loss is estimated up to $200 million per year in the turkey industry alone due to the incidence of PSE (Owens et al., 2000). As a result, a rapid chilling of carcasses is important to improve chilling efficiency and meat quality. Table 1 shows the results of carcass chilling yield and fillet cooking yield. There was no significant difference in the chilling yield and cooking yield, among chilling methods (Table 1).

**Table 1:** Measurement (%)^1^ of carcass chilling yield and fillet cooking yield (±SEM) after carcass chilling in three different methods.

Parameter	0% NaCl/0.5^o^C	4% NaCl/−2.41^o^C	8% NaCl/−5.08^o^C
Chilling yield (%)	101.4 ± 2.0	108.1 ± 7.8	102.8 ± 5.3
Cooking yield (%)	75.6 ± 1.36	73.5 ± 0.42	72.0 ± 2.41

1 Number of oberservations in each chillilng, n = 4.

After chilling, all bacteria on the turkey carcasses in subzero saline solution were significantly reduced, regardless of salt content, over the carcasses in control solution, except the MAB in 4% NaCl/−2.41°C (Table 2). Similar results were observed in broiler carcasses that were chilled in 0% NaCl/0.5°C, 3% NaCl/−1.8°C, and 4% NaCl/−2.4°C solutions, showing a stepwise bacterial reduction in *E. coli* and total coliforms, but not MAB (Lee et al., 2020). In general, Gram-negative bacteria such as *E. coli* and coliforms are labile to extreme environments such as subzero temperature, salt, high temperature, etc. (Mai-Prochnow et al., 2016; Dimitraki and Velonakis, 2007; Jay, 1992). Part of the reasons for the weak reduction in MAB is the potential of various bacterial cells including psychotropic and halophilic bacteria in addition to Gram-positive cells with the thick wall and peptidoglycan (Mackey, 2000; Tsuchido et al., 1995).

**Table 2:** Mean population (log cfu/g) (±SEM)^1^ of mesophilic aerobic bacteria (MAB), *Escherichia coli*, and total coliforms on turkey skin after chilling.

Parameter	0% NaCl/0.5^o^C	4% NaCl/−2.41^o^C	8% NaCl/−5.08^o^C

MAB	4.70 ± 0.84^a^	3.68 ± 0.11^ab^	3.34 ± 0.07^b^
*E. coli*	2.09 ± 0.33^a^	0.15 ± 0.29^b^	< 0.01 ± < 0.01
Total coliforms	1.65 ± 0.64^a^	0.15 ± 0.29^b^	< 0.01 ± < 0.01

ab Means within a raw with no common subscripts are different (*P* < 0.05).

1 Nmber of oberservations in each chillilng, n = 4.

## CONCLUSIONS

Efficient chilling of turkey carcasses is very important to improve the microbiological safety and product quality. In this study, SSC of turkey carcasses showed a significant improvement of chilling efficiency and bacterial reduction, whereas no significant difference was observed for carcass chilling yield and fillet cooking yield. Robust bacteria such as Gram-positive, halophilic, and psychotropic cells in MAB are expected to survive in subzero saline solution and contribute to a less bacterial reduction over Gram-negative cells such as *E. coli* and coliforms. It is required to further evaluate the survival of *Salmonella* and *Campylobacter* on turkey carcasses after SSC and the potential extension of red water recycle after chilling of carcasses in the unfavorable environment to bacterial growth.

## References

[bib0001] Clark D.L., Nestor K.E., Velleman S.G. (2019). Continual selection for increased 16 wk body weight on turkey growth and mat quality: 50 generation update. J. Appl. Poult. Res..

[bib0002] Dimitraki P., Velonakis E. (2007). The survival of pathogens in frozen food as a health risk. Arch. Hellenic Med..

[bib0003] Fernandez A.M., Vieira C. (2012). Effect of chilling applied to suckling lamb carcasses on hygienic, physicochemical and sensory meat quality. Meat Sci..

[bib0004] Jay J.M. (1992).

[bib0005] Kang, I. 2021. Meat processing methods (Provisional – Application # 17/249,914).

[bib0006] Kang I., Lee H.C., Adhikari B., Kwon Y.M. (2021). Effects of hot water spray and subzero saline chilling on bacterial decontamination of broiler carcasses. Poult. Sci..

[bib0007] Kang, I., and S. Hurley. 2019. Meat processing methods (US 2019/0008174 A1). Accessed Sep. 2021. https://www.patentguru.com/search?q=cpc=A23B4/09

[bib0008] Lee H.C., Erasmus M.A., Swanson J.C., Hong H.G., Kang I. (2016). Improvement of turkey breast meat quality and cooked gel functionality using hot-boning, quarter sectioning, crust-freeze-air-chilling and cold-batter-mincing technologies. Poult. Sci.

[bib0009] Lee H.C., Medellin-Lopez M., Singh P., Sansawat T., Chin K.B., Kang I. (2014). Cold-batter mincing of hot-boned and crust-frozen air-chilled turkey breast allows for reduced sodium content in protein gels. Poult. Sci..

[bib0010] Lee H.C., Metheny M.M., Viliani S., Bennett D.C., Hurley S., Kang I. (2020). Effects of subzero saline chilling on broiler chilling efficiency, meat quality, and microbial safety. Poult. Sci..

[bib0011] Lee H.C., Singh P., Strasburg G.M., Marks B.P., Jin H.W., Kang I. (2019). Comparison of meat quality and protein functionality after crust-freeze-air-chilling and cold-batter-mincing of hot-boned or chill-boned turkey fillets. Poult. Sci..

[bib0012] Mackey, B. M. 2000. Injured bacteria. In: M. Lund, T. C. Baird-Parker, & G. W. Gould (Eds.). Pages 315–341 in The Microbiological Safety, and Quality of Food. Volume 1. Aspen Publisher; Gaithersburg, MD.

[bib0013] Mai-Prochnow A., Clauson M., Hong J., Murphy M.B. (2016). Gram positive and Gram-negative bacteria differ in their sensitivity to cold plasma. Sci. Rep..

[bib0014] McKee S.R., Sams A.R. (1998). Rigor mortis development at elevated temperatures induces pale exudative turkey meat characteristics. Poult. Sci..

[bib0015] Medellin-Lopez M., Sansawat T., Strasburg G., Marks B.P., Kang I. (2014). Cold-batter mincing of hot-boned and crust-freezing air-chilled turkey breast improved meat turnover time and product quality. Poult. Sci..

[bib0016] Metheny M.M, Lee H.C., Viliani S., Bennett D., Hurley S., Kang I. (2019). Improvement of chilling efficiency and meat tenderness of broiler carcasses using sub-zero saline solutions. Poult. Sci..

[bib0017] National Turkey Federation. 2021. Turkey production by the numbers. Accessed Sep. 2021. https://www.eatturkey.org/turkeystats/

[bib0018] Owens C.M., Hirschler E.M., McKee S.R., Martinez-Dawson R., Sams A.R. (2000). The characterization and incidence of pale, soft, exudative turkey meat in a commercial plant. Poult. Sci..

[bib0019] Savell J.W., Mueller S.L., Baird B.E. (2005). The chilling of carcasses. Meat Sci..

[bib0020] Sosnicki A.A., Wilson B.W. (1991). Pathology of turkey skeletal muscle: implications for the poultry industry. Food Struct.

[bib0021] SPSS (2011).

[bib0022] Tsuchido T., Katsui N., Takeuchi A., Takano M., Shibasaki I. (1995). Destruction of the outer membrane permeability barrier of Escherichia coli by heat treatment. Appl. Environ. Microbiol..

